# The association between subjective memory complaint and objective cognitive function in older people with previous major depression

**DOI:** 10.1371/journal.pone.0173027

**Published:** 2017-03-07

**Authors:** Chung-Shiang Chu, I-Wen Sun, Aysha Begum, Shen-Ing Liu, Ching-Jui Chang, Wei-Che Chiu, Chin-Hsin Chen, Hwang-Shen Tang, Chia-Li Yang, Ying-Chin Lin, Chih-Chiang Chiu, Robert Stewart

**Affiliations:** 1 Department of Psychiatry, Cardinal Tien Hospital, New Taipei City, Taiwan; 2 Department of Psychiatry, Mackay Memorial Hospital, Taipei, Taiwan; 3 King’s College London (Institute of Psychiatry), Department of Psychological Medicine, London, United Kindom; 4 Department of Psychiatry, Cathay General Hospital, Taipei, Taiwan; 5 School of Medicine, Fu Jen Catholic University, Taipei, Taiwan; 6 Department of Psychiatry, School of Medicine, Taipei Medical University, Taipei, Taiwan; 7 Department of Psychiatry, Taipei Medical University-Wan Fang Hospital, Taipei, Taiwan; 8 Department of Psychiatry, Taipei City Hospital, Songde Branch, Taipei, Taiwan; 9 Department of Family Medicine, Taipei City Hospital, Zhongxing Branch, Taipei, Taiwan; 10 Department of Family Medicine, Shuang-Ho Hospital, Taipei Medical University, New Taipei, Taiwan; 11 Department of Family Medicine, School of Medicine, College of Medicine, Taipei Medical University, Taipei, Taiwan; Radboud University Medical Centre, NETHERLANDS

## Abstract

The goal of this study is to investigate associations between subjective memory complaint and objective cognitive performance in older people with previous major depression–a high-risk sample for cognitive impairment and later dementia. A cross-sectional study was carried out in people aged 60 or over with previous major depression but not fulfilling current major depression criteria according to DSM-IV-TR. People with dementia or Mini-Mental State Examination score less than 17 were excluded. Subjective memory complaint was defined on the basis of a score ≧4 on the subscale of Geriatric Mental State schedule, a maximum score of 8. Older people aged equal or over 60 without any psychiatric diagnosis were enrolled as healthy controls. Cognitive function was evaluated using a series of cognitive tests assessing verbal memory, attention/speed, visuospatial function, verbal fluency, and cognitive flexibility in all participants. One hundred and thirteen older people with previous major depression and forty-six healthy controls were enrolled. Subjective memory complaint was present in more than half of the participants with depression history (55.8%). Among those with major depression history, subjective memory complaint was associated with lower total immediate recall and delayed verbal recall scores after adjustment. The associations between subjective memory complaint and worse memory performance were stronger in participants with lower depressive symptoms (Hamilton Depression Rating Scale score<7). The results suggest subjective memory complaint may be a valid appraisal of memory performance in older people with previous major depression and consideration should be given to more proactive assessment and follow-up in these clinical samples.

## Introduction

Subjective memory complaints (SMC) are commonly reported by older people and often prompt concerns about the possibility of cognitive impairment expressed by patients, carers and clinicians. However, the clinical significance of SMC is still controversial. Cross-sectional studies focusing on SMC and objective cognitive impairment have shown conflicting findings, some reporting a positive association [1–5] but others reporting no association [6–9]. In longitudinal studies, SMC has been found to be associated with later cognitive decline [10] and higher incidence of dementia [11–13] although not in all studies [14, 15]. The association between SMC and incidence of dementia has been suggested to be stronger in people with less severe cognitive impairment [1, 16] but weaker in those with depression [11]. SMC is associated with a number of other states including depression [6, 8, 17–19], anxiety [17, 18], worse physical health [6, 20] and personality traits, such as neuroticism [6, 21]. Inconsistencies may arise in part because of different SMC measures [1, 22], different sample populations, limited cognitive tests, and failure to take depression into consideration [1, 21].

Major depression in older people often causes impairment in different cognitive domains, including information processing, memory, and executive functioning. These difficulties may continue to persist even after improvement in depression symptoms [23, 24]. In euthymic older people with previous major depression, it has been reported that 52.3 percent met the definition of mild cognitive impairment (MCI) [25], SMC being one of its component criteria [26]. Indeed, people with late-life depression are at an increased risk of dementia [27]. The cognitive impairments, comorbid physical problems, and frequent multiple somatic complaints, including memory complaints, in people with late-life depression make the relationship between depression, SMC and objective cognitive impairment in this specific group more complicated. Whether SMC is a valuable indicator for objective impairment in older people with major depression is still unclear.

In this study, we enrolled older people with history of major depression and healthy controls (older people without history of depression) to investigate associations between SMC and objective cognitive performance in older people with previous major depression, and to compare their cognitive function with healthy controls.

## Materials and methods

This cross-sectional study drew samples of older people with previous depression from the outpatient psychiatric services of four hospitals in Taipei and healthy controls from the Department of Health Management Center, which provides a health check program for the general population. The study received full approval from the institutional review boards of Taipei City Hospital, Cathay General Hospital, Mackay Memorial Hospital, and Taipei Medical University-Wan Fang Hospital, and was designed primarily to test associations between mental health outcomes and levels of polyunsaturated fatty acids in older people with previous major depression [28, 29]. The analysis reported here took advantage of this informative ‘at risk’ sample to conduct a secondary analysis with a different objective but one which was conceived at the start of the study.

### Participants

One hundred and fifty-nine participants, 113 older people with previous major depression and 46 healthy controls, were enrolled in this study. Potential participants with previous depression were screened and selected according to the following criteria: (1) age ≧60 years; (2) a previous diagnosis of major depressive disorder according to the Structured Clinical Interview for DSM IV-TR Axis-I Disorder (SCID); (3) relatively stable clinical condition over the previous three consecutive weeks judged by referring psychiatrists [30] and no longer fulfilling current diagnosis of major depression by SCID; (4) capacity to provide informed consent. Exclusion criteria were: (1) severe or acute medical illness within 3 months preceding the study; (2) neurological disorders such as delirium, Parkinson’s disease, aphasia or multiple sclerosis; (3) dementia or a Mini-Mental State Examination (MMSE) score below17 [31]; (4) alcoholism defined by the Alcohol Use Disorders Identification Test score≧8 in males or ≧6 in females [32]; and (5) electroconvulsive therapy in the preceding year. Inclusion criteria for the healthy comparator group were as follows:(1) age≧60 years; (2) no current or previous diagnosis of major depression or any other psychiatric diagnosis by SCID; (3)17-item Hamilton Depression Rating Scale (HDRS)<7; (4) none of the abovementioned exclusion criteria; (5) capacity to provide informed consent. All eligible potential participants in the outpatient psychiatric services or those in the Department of Health Management Center were invited to take part in this study. After full explanation of the study, two research psychiatrists (S.C.L. and C.C.C.) did an interview for SCID and for evaluation of capacity of providing informed consent. All participants provided written informed consent before entering the study.

### Study design and measurements

A cross-sectional study was carried out. SMC was assessed using questions from the Geriatric Mental State schedule (GMS) [33], which includes an evaluation of the presence and severity of self-reported memory difficulties, recent forgetfulness of names, misplacing objects and effort to remember things. SMC was defined on the basis of a score ≧4 on this GMS sub-scale (which has a maximum score of 8), a cut-off point applied in previous research using an identical scale [20, 34]. In addition, the 17-item HDRS and MMSE were administered.

Participants were administered a series of cognitive tests and a structured questionnaire. SMC was ascertained after cognitive assessment, i.e. the examiner did not know SMC status at the time of cognitive examination. Cognitive tests were selected to cover a broad range of domains, with acceptable psychometric properties for this specific group and availability in Chinese. They comprised the following: (1) A measure of verbal memory using the word list subtest of Wechsler Memory Scale-III (WMS-III); (2) Attention and psychomotor speed was assessed by Color trail test-1 (CTT-1) and WAIS-III digit symbol substitution test; (3) Visuo-spatial processing was measured by the WAIS-III block design; (4) Cognitive flexibility, a measure of executive function was assessed with the CTT-2; (5) Semantic verbal fluency was assessed using fruit naming.

A structured questionnaire was used to collect demographic and health-related factors, including age, gender, years of education, and physical health problems. History of the following symptoms/disorder was enquired about in addition to SMC: angina, previous stroke, arthritis, asthma, bowel problems, cough, diabetes, poor eyesight, headaches, poor hearing, previous heart attack, hypertension, skin problems and insomnia [34, 35]. The sum of the total number of symptoms/disorders, representing physical health status, was calculated. Three categories (≦3, 4–5, or ≧6) were originally applied based on previous work [20], but this was condensed into a binary variable(≦3, ≧4) due to an insufficient number of cases (n = 7) in the third category. Age of onset for major depression, onset of depression before 60 year-old (early-onset depression) vs. onset after 60 year-old (late-onset depression), was confirmed by structured interview and chart review if feasible.

Blood samples were assayed by workers blind to the study design and other measurements. A modified polymerase chain reactions was used to identify apolipoprotein E (ApoE) genotype, re-categorised as a binary variable based on the presence or not of at least one ε4 allele [36].

### Statistical analyses

The following eight cognitive parameters were selected for final comparison based on their theoretical representativeness of cognitive domains, previous research, and observed sensitivity to age and education [28]: total immediate recall, delayed recall, and recognition from word list test of WMS-III, CTT-1 completion time and CTT-2 completion time from CTTs, block design score, verbal fluency score, and digit symbol substitution test score. Their representativeness was further confirmed by factor analysis.

At first, the main covariates were compared between the three groups, elderly with previous depression and SMC, elderly with previous depression but without SMC, and the healthy controls, using ANOVA to assess differences in continuous variables and chi-square tests for categorical variables. Estimated individual cognitive test scores were displayed for each group after adjustment for age, gender, and education. Multiple linear regressions were then separately performed to examine the associations between individual cognitive parameter score (dependent variable) and the three groups by adjustment for age, gender, education, presence of the ApoE ε4 allele, and other potential confounders (variables affecting standardized β in univariate analysis of study interest at least 15%). Exploratory stratification was further carried out for higher or lower cognitive function levels (the latter being defined as an MMSE score of 26 or below) [16] and higher or lower current depressive symptoms (the latter being defined as a HDRS score of 6 or below) [37] for those cognitive parameters differing between SMC and non-SMC group. The interactions of SMC with higher/lower cognitive function and higher/lower depressive symptoms for the individual cognitive parameter were tested. Data were analyzed by STATA 9.0 (Texas, USA) and the statistical significance was defined if *p*-values were smaller than 0.05 (two-sided).

## Results

Characteristics of the three groups (elderly with previous depression and SMC, elderly with previous depression but without SMC and healthy controls) are compared in Table 1. SMC was present in more than half of the participants with depression (55.8%). The mean MMSE score was 27.2 ± 2.2 (range 17–30) with median 28. Compared to participants with previous depression, healthy controls were older, more often male, more educated, and had higher MMSE score, fewer depressive symptoms, and fewer physical health problems. Within the group with previous depression, those reporting SMC had more depressive symptoms, lower MMSE score, and more physical health problems compared to those without SMC. There were no other statistically significant differences in other demographic and clinical characteristics between SMC and non-SMC depression groups. Among older people with previous depression, 53.1% of them were early-onset depression, while 46.9% patients were late-onset depression. The time period from the previous depressive episode in participants with previous depression (n = 103) ranged from 1–228 months (mean ± S.D: 29.7± 49.2 month), and medium was 12 months with interquartile range from 2 to 36 months. There was no difference between SMC and non-SMC groups in terms of the proportions of early onset depression or with less than a one-year interval since the previous depression (Table 1).

**Table 1:** Demographic and clinical characteristics of participants.

	Participants with previous depression		Healthy controls (n = 46)	*p*-value^2^	Cohen’s *d*	
					(95%C.I.)	
	SMC^1^ (n = 63)	Non-SMC (n = 50)			(*d*_*1*_)^3^	(*d*_*2*_)^*4*^
**Age, mean (SD**^**5**^**) years**	66.7 ± 6.2	66.6 ± 5.7	69.0 ±6.8	0.110	0.02	0.36
					(-0.35~0.39)	(-0.74~0.02)
**Gender (% of female)**	71.4	76.0	37	*<*0.001	<0.001	<-0.001
**Education, mean (SD) years**	10.0 ± 5.1	11.3 ± 3.6	12.2 ± 4.4	0.020	-0.29	-0.46
					(-0.66~0.08)	(-0.84~-0.07)
**Hamilton depression rating scale score, mean (SD)**	7.4 ± 3.8	4.7 ± 3.4	1.9 ± 1.8	*<*0.001	0.74	1.18
					(0.36~1.13)	(1.31~2.21)
**Physical health (number of symptoms), mean (SD)**	3.0 ± 2.0	1.8 ± 1.5	2.0 ± 1.6	0.001	0.67	0.54
					(0.29~1.05)	(0.16~0.93)
**Percentage with 4 or more physical diseases (%)**	33.9	12.2	15.6	0.010	-0.03	-0.02
**Mini-Mental State Examination score, mean (SD)**	26.8± 2.5	27.7 ± 1.7	28.0 ± 1.6	0.003	-0.41	-0.55
					(-0.79~-0.04)	(-0.94~-0.17)
**Early onset depression**^**6**^ **n (%)**^**7**^	36(58.1)	24(48.0)		0.288	-0.002	
**The period from previous depression≦12 months n (%)**^**8**^	27(49.1)	26(54.2)		0.607	0.001	
**Percentage of possessing ApoEε4 allele**^**9**^ **(%)**	11.5	16.0	13.5	0.790	0.015	<0.001
**Percentage of medication use**						
**Cardiovascular drugs n (%)**	31(67.4)	15(30.0)		0.032	-0.010	
**Gastrointestinal drugs n (%)**	18(29.0)	9(18.0)		0.175	-0.012	
**Endocrine drugs n (%)**	9(14.3)	8(16.0)		0.800	0.004	
**Hypnotic drugs n (%)**	55(87.3)	40(80.0)		0.292	<-0.001	
**Antipsychotic drugs n (%)**	9(14.3)	8(16.0)		0.800	0.004	
**Antidepressants n (%)**	54(85.7)	41(82.0)		0.592	<-0.001	
**Tricyclic antidepressants n (%)**	12(19.4)	3(6.0)		0.039	-0.071	
**Trazodone n (%)**	12(19.0)	12(24.0)		0.523	0.007	
**SSRI**^**10**^ **n (%)**	29(46.0)	29(58.0)		0.206	0.003	
**SNRI**^**11**^ **n (%)**	7(11.1)	8(16.0)		0.447	0.017	
**Other antidepressants n (%)**	9(14.3)	6(12.0)		0.722	-0.008	

^1^SMC: subjective memory complaints.

^2^*p*-value for the results of comparison between the three groups (ANOVA) for continuous variables and chi-square test for category variables.

^3^*d*_*1*_: Cohen’s d, comparison of SMC and non-SMC depression group.

^4^*d*_*2*_: Cohen’s d: comparison of SMC depression group and healthy controls.

^5^SD: standard deviation.

^6^Early onset depression: age onset of depression less than 60 year-old.

^7^n = 112.

^8^n = 103.

^9^Participants possessed at least one allele of ApoE4.

^10^SSRI: Selective Serotonin Reuptake Inhibitors.

^11^SNRI: Selective serotonin-norepinephrine reuptake inhibitors.

There were 84.1% of participants taking antidepressants while receiving assessment. The most frequent antidepressants prescribed were selective serotonin reuptake inhibitors (51.3%), and followed by trazodone (21.2%), selective serotonin-norepinephrine reuptake inhibitors (13.3%), tricyclic antidepressants (13.3%), and other antidepressants (13.3%). Comparisons of antidepressant use between SMC and non-SMC depression groups are displayed in Table 1. The SMC depression group had a higher percentage of tricyclic antidepressant use compared to non-SMC depression group, but there were no other significant differences. The other medications taken by participants with previous depression are also shown in Table 1, restricting this to medication use present in more than 10% of the SMC or non-SMC depression groups. The SMC group had higher percentage use of cardiovascular drugs compared to the non-SMC group. However, it did not have a substantial effect on the associations between SMC and immediate or delayed recall score.

Table 2 displays the estimated scores for the eight selected cognitive test parameters in the three groups after adjustment for age, gender, and education. It also shows the results of multiple linear regressions to investigate the differences of individual cognitive parameter score between the three groups after adjustment for age, gender, education, depressive symptoms, physical problems, tricyclic antidepressant use and ApoE ε4. In the analysis, the scores of recognition, verbal frequency and block design were similar between the three groups. Participants with history of major depression (including SMC and non-SMC groups) had significantly worse performance on completion time for CTT-1, CTT-2, and digit span substitution test compared to the healthy controls. Compared to the non-SMC group with previous depression, the participants with SMC and previous depression showed significantly worse performance only in total immediate recall and delayed recall among these eight cognitive parameters. Healthy controls did better than the SMC group in these two cognitive parameters but were not significantly different from the non-SMC group. If we excluded two participants fulfilled the diagnosis of amnesic MCI [38], the main results are the same. The final regression models using immediate recall score and delayed recall score as dependent variable separately to assess their associations with SMC showed that after adjustment for potential confounders standardized *β* coefficient (SMC group vs. non-SMC group) was -0.27 and -0.24, respectively. Considering their potential effects on cognition, the final model was further adjusted for age of onset and the time period from the last depressive episode separately. However, they did not show substantial effects on the standardized *β* coefficient (-0.25 and -0.27 for immediate recall score and -0.24 and -0.25 for delayed recall score).

**Table 2:** Comparisons of cognitive test scores between SMC^1^ and non-SMC group of elderly with previous depression and healthy controls.

Cognitive test parameters	Elderly with previous depression(Mean^2^±SE^3^)		Healthy controls Mean^2^±SE^3^(n = 46)	F value	Adjusted R^2^	Cohen’s *d*		Post hoc comparison
						**(95%C.I.)**		
	SMC group (n = 63)	Non-SMC group (n = 50)		(*p*-value)^6^		(*d*_1_)^7^	(*d*_2_)^8^	
**CTT-1(sec)**^**4**^	69.5±3.2	69.4±3.5	50.4±3.8	*F*(7,135) = 9.34	0.291	0.69	0.09	SMC>controls; non-SMC>controls
				(*p*<0.001)		(0.30~1.09)	(-0.28~0.46)	
**CTT-2(sec)**	142.5±5.0	142.0±5.5	109.3±6.0	F(7,135) = 8.78	0.277	0.94	0.10	SMC>controls; non-SMC>controls
				(*p*<0.001)		(0.54~1.34)	(-0.27~0.47)	
**Total immediate recall**	23.8±0.6	27.2±0.7	29.1±0.7	F(7,135) = 10.85	0.327	-0.95	-0.78	Controls>SMC; non-SMC>SMC
				(*p*<0.001)		(-1.35~-0.55)	(-1.16~-0.40)	
**Delayed recall**	4.9±0.3	6.2±0.3	6.9±0.4	F(7,135) = 6.78	0.222	-0.86	-0.64	Controls>SMC; non-SMC>SMC
				(*p*<0.001)		(-1.26~-0.46)	(-1.02~-0.26)	
**Recognition**	20.8±0.3	21.6±0.4	22.1±0.4	F(7,135) = 3.08	0.093	-0.66	-0.36	No any difference between groups
				(*p* = 0.005)		(-1.05~-0.27)	(-0.74~0.01)	
**Verbal Fluency**	13.4±0.3	12.9±0.4	14.8±0.4	F(7,135) = 6.5	0.212	-0.37	0.06	Controls>non-SMC
				(*p*<0.001)		(-0.76~-0.01)	(-0.31~0.43)	
**DSST**^**5**^	44.0±1.4	43.8±1.6	55.3±1.7	F(7,135) = 24.90	0.541	-0.79	-0.15	Controls>non-SMC; Controls>SMC
				(*p*<0.001)		(-1.18~-0.39)	(-0.53~0.22)	
**Block design**	26.6±1.1	25.4±1.2	29.2±1.3	F(7,135) = 7.40	0.240	-0.47	0.03	No any difference between groups
				(*p*<0.001)		(-0.85~-0.08)	(-0.34~0.40)	

^1^SMC: subjective memory complaints.

^2^The data presented here are the estimated value of cognitive test scores after adjusted for age, gender, and education.

^3^SE: standard error.

^4^CTT: Color Trail Test.

^5^DSST: Digit Symbol Substitution Test.

^6^All F values (*p*-values) presented here derived from separate linear regression models with the three groups (SMC, Non-SMC, Controls) entered against individual cognitive parameter scores as the dependent variables after adjustment for age, gender, education, physical problems, Hamilton Depression Rating Scale scores and ApoE4.

^7^*d*_1_: The comparison of Cohen’s d with SMC group and controls.

^8^*d*_2_: The comparison of Cohen’s d with SMC group and Non-SMC group.

Since only immediate recall and delayed recall scores were found to differ significantly between the SMC and non-SMC groups, participants with previous depression were stratified by cognitive function and depressive symptoms to investigate potential effect modification (Table 3). On stratification, the associations between SMC and worse total immediate recall or delayed recall were only significant in participants with lower depressive symptoms (HDRS<7) and the *p*-values for the interaction terms between SMC and depression for immediate recall and delayed recall outcomes were 0.049 and 0.02, respectively. The association between SMC and worse immediate recall was only significant in participants with lower cognitive function (MMSE≦26) while neither higher nor lower cognitive function group of participants with previous depression showed significant association between SMC and worse delayed recall. The *p*-values for the interaction terms between SMC and higher/lower cognitive function for immediate recall and delayed recall were 0.003 and 0.29, respectively.

**Table 3:** Multiple linear regression models for associations between SMC and scores of immediate recall and delayed recall stratified by cognition or depression in elderly with previous depression^1^^,^^2^.

Stratification	Association between SMC and scores of immediate recall and delayed recall (B-coefficient, *p*-value)^3^^,^ ^4^^,^^5^					
	Immediate recall score			Delayed recall score		
	B-coefficient (95% C.I.)	standardized *β*	*p value*	B-coefficient (95% C.I.)	standardized *β*	*p value*
**High cognitive function(MMSE>26; n = 66)**	-1.28 (-4.08~1.51)	-0.12	0.361	-1.13(-2.47~0.21)	-0.22	0.096
**Low cognitive function(MMSE≦26; n = 40)**	-5.31(-8.65~-1.97)	-0.50	0.003	-0.40(-2.23~1.43)	-0.09	0.662
**High depressive symptoms(HDRS**^**6**^**≧7; n = 44)**	-1.76(-5.13~1.62)	-0.14	0.298	-0.10(-1.69~1.50)	-.018	0.903
**Low depressive symptoms(HDRS<7; n = 62)**	-3.96(-6.67~-1.24)	-0.34	0.005	-1.27(-2.59~-0.40)	-0.23	0.057

^1^SMC: subjective memory complaints.

^2^High and low cognitive function were defined as MMSE**>**26 and MMSE**≦**26.

^3^All the B coefficients (*p* values) presented here derived from separate linear regression models with SMC entered against score of total immediate recall or delayed recall as the dependent variables.

^4^The multiple regression models were adjusted for age, gender, education, physical problems, ApoE4, Hamilton depression ratingscale score and tricyclic antiderpessant use.

^5^The value of B coefficient means the differnece of memory test score between SMC group and non-SMC group after adjustment.

^6^HDRS: Hamilton Depression Rating Scale.

## Discussion

The principal findings of this study were that in older people with previous major depression SMC was significantly associated with objective memory impairment, specifically in immediate recall and delayed verbal recall although both SMC and non-SMC group with previous depression had worse performance in tests of attention, executive function, and memory compared to healthy controls. The associations of SMC with worse memory performance were stronger in those with lower depressive symptoms in this specific sample.

To the best of our knowledge, this is the first study to investigate associations between SMC and objective cognitive function specifically in older people with previous depression. Unsurprisingly, participants with a history of major depressive disorder had worse physical health and depressive symptoms, and lower cognitive function than healthy controls. In participants with previous depression, those with SMC had worse physical health and more depressive symptoms, consistent with previous studies in community samples [6, 8, 19, 20]. Indeed, the physical illness has substantial effects, decreasing the standardized β by about 15% for the association between SMC and memory performance. Thus, it was entered into final regression models.

Compared to healthy controls, participants with a history of depression (both with/without SMC), had worse performance on attention (completion time for CTT-1), executive function (CTT-2, digit span substitution test) and episodic memory (scores of total immediate recall and delayed recall). These are consistent with previous findings suggesting that older people with depression have relative impairment in domains of attention, episodic memory, processing speed, visuo-spatial function and executive ability [23, 39, 40]. Among participants with previous depression, the most significant impairments of objective cognitive function associated with SMC group were in immediate and delayed recall, and these associations persisted after adjustment for potential confounders. In unselected community samples, contemporaneous associations between SMC and objective memory impairment have been inconsistent [1–7, 9]. Although there have been some reports of associations between SMC and impairments in other cognitive tests such as verbal fluency [8], we found no such association in people with previous depression. Compared to other cognitive domains, episodic memory impairment in particular has been found to be associated with a substantial and persistent elevated risk of developing Alzheimer dementia in people with MCI or SMC [41, 42]. Our findings therefore suggest that SMC may represent valid memory appraisal rather than a complaint related to depression in this specific population.

While a number of studies have concluded that SMC is more influenced by depressive symptoms than actual cognitive impairment [17, 43], these results may be biased by heterogeneity between samples and studies, lack of a “gold standard” validated measurement of SMC, or inadequate measurement of depressive symptoms [44, 45]. After adjustment for depression, however, our study still found a relationship between SMC and memory impairment–both immediate and delayed recall–in participants with a history of depression. The result is compatible with the most recent meta-analysis of cross-sectional findings that subjective cognitive complaints were associated independently with both objective cognitive function and depressive symptoms [44]. In older people with previous depression, both depressive symptoms and objective cognitive function may play a role in the association of SMC with true cognitive impairment as shown by the stratified analysis. We suggest that older people with previous depression but still having some residual depressive symptoms may have frequent negative self-appraisal and somatic concerns resulting in more SMCs. On the other hand, this population is at higher risk of cognitive impairment, MCI or even dementia. Therefore true objective memory impairment may also contribute to their memory complaints, which was supported by our findings that participants with lower MMSE had stronger associations of interest. Taking all the aforementioned findings into consideration, we believe that both depressive symptoms and memory impairment may play a crucial role in the presentation of SMC. Therefore, in clinical practice, the presence of SMC in elderly with previous depression requires a thorough evaluation: not just for depressive disorders but also underlying or comorbid cognitive impairment.

Several hypotheses linking depression and cognitive impairment have been proposed, including hypercorticoid-related hippocampal dysfunction, cardiovascular disease and ischemic white matter hyperintensities (WMH), and depression as an early symptom of dementia [46]. Although vascular risk factors were only weakly associated with SMC in a cross-sectional study [47], the frequency of severe WMH has been found to be higher in people with SMC [48]. In addition, SMC has been reported to be associated with smaller hippocampal volume [49–52] and higher amyloid-β burden in PET imaging [53], which suggest that SMC may be emerging at quite early stages of cognitive decline. Thus, SMC may need special attention and careful assessment, particularly where other risk factors are present.

Strengths of the study include the ascertainment of SMC from a structured questionnaire, the comprehensive cognitive assessment, and screening of potential participants with a structured diagnosed interview to confirm a previous diagnosis of major depression. We acknowledge some limitations. First, the sample size was relatively small. Second, the participants were recruited from psychiatric outpatient services rather than being community-based, although this was necessitated by our intended focus on this particular population (older people with previous major depression). Third, the healthy controls were not representative of the general community, since they were enrolled from volunteers who participated in a health check program. Fourth, we did not adjust for multiple comparisons in the analysis because we were primarily looking for consistency across different assessments and aiming to focus on findings which could be demonstrated to be consistent. Fifth, MMSE score of 17 was relatively lower to be used as a cut-off score, as applied elsewhere [31]. Two included participants fulfilled a diagnosis of amnestic MCI; however, the main results were not changed if the two cases were deleted. Finally, the study was cross-sectional in design and direction of causation cannot be inferred.

In summary, we found that there was a significant association between SMC and impairment of immediate recall and delayed recall in older people with previous major depression, a group known to have higher prevalence of MCI and to have a raised risk for further dementia. The results suggest that SMC may be a valid symptom of memory impairment in this group. Since they are often known to clinical services, there may be indications for more careful cognitive assessment and follow-up where memory complaints are mentioned.
